# Doctors and Colonial Income Tax

**Published:** 1924-01

**Authors:** 


### Doctors and Colonial Income Tax.

SIR,—A great deal of curious information is presented in a book just issued by Somerset House regarding the regulations of different British Dominions on the subject of income tax. It is not usual in such legislation to find special provisions regarding specified occupations, and one is not surprised to see in the great majority of the codes no particular enactments in favour of or against the medical profession.

But there are exceptions. These are two in number, and only one of them directly applies to doctors. That is in the African possession known as the Tanganyika Territory. It must have progressed far along the pathway of civilization, for it has an income tax law of its own. It may be inferred that doctors are at a premium there, for the attraction is held out to them that they will be charged no income tax on their professional gains. They must, of course, be duly qualified, which we take to be what is meant by "licensed." The exemption applies to medical, surgical and dental services, and the sale of drugs and appliances, but only to patients. The exemption has no limit. It is, however, rather a pity to see that the framers of this legislation, echoing presumably the practice of those for whom they were legislating, express themselves, not in pounds or guineas, but in shillings. Thus the general exemption limit for other classes is fixed at 2,000 shillings. It is permissible to imagine that this may be suggestive.

The next Dominion to which we pass is Victoria. If the Tanganyika exemption could be transported to that Australian State, it would be worth while. But all that is found in the Victorian code is a clause to the effect that certain medical, etc., expenses may be passed as deductions from income before reaching the taxable figure. There are payments in respect of the illness of the taxpayer himself, his wife, or minor child, to any legally qualified medical practitioner or any public or private hospital, any nurse or any chemist. Unless the expenditure on any head exceeds £2 for one illness, it is reckoned too trifling to be considered. Dental treatment is not specified. These allowances are excluded if the income exceeds £800. There is also an allowance of £20 for funeral of wife or minor child, but not of the taxpayer himself. All these, so far as I know, are novelties in income tax law. W. S. E.

